# Data-Driven Health Prognostics of NMC Lithium-Ion Batteries via Impedance Spectroscopy Using a Hybrid CNN-BiLSTM Model

**DOI:** 10.3390/s26082492

**Published:** 2026-04-17

**Authors:** Zhihang Liu, Kai Fu, Jiahui Liao, Ulrich Stimming, Donghui Guo, Yunwei Zhang

**Affiliations:** 1 School of Physics, Sun Yat-sen University, Guangzhou 510275, China; 2 Guangdong Provincial Key Laboratory of Magnetoelectric Physics and Devices, Sun Yat-sen University, Guangzhou 510275, China; 3 The Faraday Institution, Quad One, Becquerel Avenue, Harwell Campus, Didcot OX11 0RA, UK

**Keywords:** electrochemical impedance spectroscopy, lithium-ion batteries, state of health estimation, remaining useful life prediction, deep learning, neural networks

## Abstract

Accurate and robust battery health prognostics are critical for reliable battery management in electronic devices and electric vehicles. Previous studies have demonstrated that combining electrochemical impedance spectroscopy (EIS) with machine learning enables accurate health-state forecasting in LiCoO_2_ coin cells. However, the applicability of this EIS-AI paradigm across diverse chemistries and industrial-grade battery formats remains unvalidated, limiting its practical deployment in energy storage systems. Here, we develop an EIS–AI battery prognostic framework and validate its performance on LiNi_1/3_Mn_1/3_Co_1/3_O_2_ (NMC111) cylindrical cells and LiNi_0.8_Mn_0.1_Co_0.1_O_2_ (NMC811) pouch cells. A hybrid Convolutional Neural Network–Bidirectional Long Short-Term Memory (CNN–BiLSTM) architecture is developed to estimate state of health (SoH) and predict remaining useful life (RUL) from EIS spectra. Trained on an in-house dataset comprising over 13,000 impedance spectra from 22 cells (8 NMC111 and 14 NMC811), the model achieves robust performance, with average coefficients of determination ($R^{2}$) exceeding 0.92 for SoH estimation and 0.90 for RUL prediction across various batteries and cycling protocols. Salient feature analysis further reveals chemistry- and protocol-dependent frequency regimes associated with degradation. These results demonstrate that impedance spectra constitute physically informative descriptors for data-driven battery prognostics and provide a scalable and interpretable pathway for deploying EIS-AI frameworks in real-world battery management systems (BMSs).

## 1. Introduction

Rechargeable lithium-ion batteries underspin modern energy technologies, powering applications from portable electronics to electric vehicles owing to their high energy density [1,2,3], long cycle life, and cost efficiency [1,2,3,4,5]. However, their long-term reliability is constrained by coupled electrochemical and mechanical degradation processes [4,5,6,7]. Once the capacity falls below a critical threshold, the battery can no longer sustain required performance and may pose safety risks [8]. Accurate assessment of battery health, typically through the estimation of state of health (SoH) [9] and prediction of remaining useful life (RUL) [10], is therefore essential to ensure reliability and safety. However, degradation in lithium-ion batteries is governed by highly nonlinear and interdependent mechanisms [11] influenced by chemistries, cell formats and operating conditions [12], making accurate battery health diagnostics and particularly prognostics challenging.

Traditional approaches for forecasting battery performance rely on empirical or physics-based models [13] that simulate individual degradation phenomena, such as solid–electrolyte interphase (SEI) growth [14,15,16], lithium plating [17,18,19], active material loss [20,21], and impedance rise [22]. While these approaches provide valuable mechanistic insights, modeling each degradation process separately limits scalability and often fails to capture their coupled aging effects under realistic operating conditions. Moreover, accurate parameter calibration requires extensive domain expertise, restricting their general application in battery management systems (BMSs).

Data-driven approaches have emerged as powerful tools for battery diagnostics and prognostics by identifying statistical correlations between measurable signals and degradation indicators without explicitly modeling degradation mechanisms [23,24,25,26]. By leveraging historical cycling datasets and machine learning (ML) algorithms, such methods enable accurate estimation of SoH and forecasting of RUL. For instance, variance features [27] extracted from discharge voltage and capacity curves have been used to predict end of life (EoL) via elastic net models, and localized incremental-capacity features [28] combined with Gaussian process regression (GPR) have been employed for SoH estimation. Deep learning networks trained on voltage, current, and temperature data have also demonstrated strong RUL forecasting performance [29]. However, these capacity- or voltage-based features primarily capture macroscopic cycling behaviors and are often sensitive to measurement noise. More importantly, they provide limited insight into the underlying electrochemical degradation processes and may struggle to provide accurate prediction of battery systems with complex degradation patterns.

In contrast, electrochemical impedance spectroscopy (EIS) provides frequency-resolved information associated with charge-transfer kinetics, ion diffusion, and interfacial reactions [30,31,32], offering a physically interpretable and information-rich basis for data-driven battery prognostics. Conventional EIS analysis relies on equivalent circuit model fitting [33,34], where parameter identification is nonunique [35,36] and difficult to generalize across chemistries and operating conditions. To address these limitations, EIS-AI approaches have also been explored for battery prognostics. Nevertheless, many of these methods still rely on handcrafted features [37,38,39] or equivalent-circuit-derived parameters as model inputs [40,41,42], which require domain knowledge and may introduce human bias into feature selection. More recent studies have shown that directly using raw impedance spectra as input to ML models can bypass explicit circuit fitting and achieve accurate battery health prediction, including SoH estimation and RUL prediction, with representative demonstrations in LiCoO_2_ coin cells [43,44,45,46,47]. These findings highlight the promise of combining EIS with AI for battery health forecasting. However, whether this raw-spectrum-based strategy can be generalized to more practically relevant battery systems—particularly high-performance NMC chemistries and industrial-grade cylindrical and pouch cells under heterogeneous operating conditions—has not yet been comprehensively validated. Addressing this gap is critical for practical integration of EIS-AI battery prognostic frameworks into real-world BMSs.

Here, we develop and experimentally validate a raw-EIS-based AI prognostic framework for accurate, interpretable, and scalable battery health forecasting across multiple chemistries, cell formats and protocol-dependent degradation pathways. A comprehensive in-house dataset is constructed, comprising over 13,000 impedance spectra from 8 LiNi_1/3_Mn_1/3_Co_1/3_O_2_ (NMC111) cylindrical cells and 14 LiNi_0.8_Mn_0.1_Co_0.1_O_2_ (NMC811) pouch cells cycled under three distinct operating protocols. Leveraging this dataset, we implement a hybrid deep-learning architecture combining convolutional neural networks and bidirectional long short-term memory networks (CNN-BiLSTM) for both SoH estimation and RUL prediction from EIS spectra. The proposed framework achieves average coefficients of determination ($R^{2}$) values exceeding 0.92 for SoH estimation and 0.90 for RUL prediction across both battery systems, outperforming conventional models in accuracy and robustness. Notably, the model maintains robust predictive performance across cells with distinct degradation pathways under diverse cycling conditions. Salient feature analysis further reveals diffusion-dominated aging in NMC111 and protocol-dependent interfacial processes degradation in Ni-rich NMC811, providing mechanistic insight into the impedance-derived features. These results establish raw EIS as a broadly applicable degradation descriptor for data-driven battery prognostics and demonstrate the potential of EIS-AI frameworks for real-time health monitoring in next-generation BMSs.

## 2. Materials and Methods

### 2.1. Data Generation

Cycling experiments were conducted on 8 fresh commercial Sony NMC111 18650 cylindrical cells (Sony Energy Devices Corporation, Minato-ku, Tokyo, Japan) and 14 fresh NMC811 A7 balanced full pouch cells (7.5 cm × 10.5 cm) manufactured on the WMG battery scale-up pilot line (WMG, University of Warwick, Coventry, UK) [48]. The nominal capacities of the NMC111 and NMC811 cells were 680 mAh and 200 mAh, respectively. Both cell types employed graphite as the negative electrode.

The 8 NMC111 cylindrical cells (marked as NMC111_01-NMC111_08) underwent galvanostatic cycling at room temperature (25 °C ± 2 °C) under a mildly accelerated aging protocol: 3 C-rate charge (2.04 A) to 4.2 V and 1.5 C-rate discharge (1.02 A) to 3.0 V. This protocol promotes measurable degradation while maintaining electrochemical stability. The 14 NMC811 pouch cells were also cycled at room temperature (25 °C ± 2 °C) but under three distinct current–voltage conditions to induce varied degradation pathways, enabling evaluation of the robustness and accuracy of the proposed EIS-AI framework across diverse operating protocols. An overview of the cell groups and their corresponding cycling conditions is provided in Table 1. Group I (3 cells marked as NMC811_01-NMC811_03) represents the baseline condition, Group II (8 cells marked as NMC811_04-NMC811_11) applies an extended upper cut-off voltage to accelerate high-voltage degradation, and Group III (3 cells marked as NMC811_12-NMC811_14) uses a higher current rate within the standard voltage window to probe the effect of increased current density.

Galvanostatic cycling measurements were performed using an Ametek Parstat MC PMC-1000 system (AMETEK, Inc., Oak Ridge, TN, USA). The cells were held in custom-made fixtures and connected in a four-wire configuration. The fixtures were calibrated using Ametek’s AC dummy cell prior to testing, and the cells together with the fixtures were placed in Memmert HPP-110 climate chambers (Memmert GmbH + Co. KG, Schwabach, Germany) to ensure temperature-controlled operation.

EIS measurements were performed during every even-numbered cycle over a frequency range of 0.02 Hz–20 kHz, with 10 points per octave and a 5 mA excitation current. For the NMC111 dataset, the EIS spectra used in this work were collected after charging to 100% SoC followed by a 15 min rest. For the NMC811 dataset, spectra were additionally collected under four designated states: immediately after charging to 100% SoC (state I), after charging to 100% SoC followed by a 15 min rest (state II), immediately after discharging to 0% SoC (state III), and after discharging to 0% SoC followed by a 15 min rest (state IV). Capacity loss was determined after every odd-numbered cycle. All EIS measurements were carried out in galvanostatic mode using a Bio-Logic VMP-3 electrochemical workstation (Bio-Logic Science Instruments, Seyssinet-Pariset, France) with a four-wire configuration. Prior to data acquisition, the instrument was calibrated using the manufacturer’s standard dummy cell (DC3; Bio-Logic Science Instruments, Seyssinet-Pariset, France). The measurement setup was further checked using a short-circuit dummy to confirm negligible contact resistance and no obvious parasitic capacitive contribution from the setup.

### 2.2. CNN-BiLSTM Architecture

A hybrid CNN–BiLSTM architecture is employed to characterize the nonlinear and long-term degradation behavior of NMC111 and NMC811 batteries from impedance data. The model is structured to capture both frequency-resolved spectral characteristics and their progressive evolution during cycling. At each cycle t, the input consists of a fixed-length impedance vector derived directly from EIS measurements. For each of the 60 frequency points (0.02 Hz–20 kHz), both the real and imaginary components of the complex impedance are retained, yielding a 120-dimensional feature vector:

(1) $$x_{t}=\left[Re\left(Z\left(f_{1}\right)\right),\ldots ,Re\left(Z\left(f_{60}\right)\right),Im\left(Z\left(f_{1}\right)\right),\ldots ,Im\left(Z\left(f_{60}\right)\right)\right]\in R^{120},$$

where $Z(f_{i})$ denotes the complex impedance at frequency $f_{i}$.

Since all spectra are collected on the same frequency grid, no additional resampling or interpolation is required. For constructing the SoH and RUL labels, the capacity sequences are smoothed using a Savitzky–Golay procedure to reduce the influence of measurement fluctuations. Before being fed into the CNN-BiLSTM architecture, both the impedance features and the labels are independently normalized using Min–Max scaling:

(2) $$x^{norm}=\frac{x-x_{min}}{x_{max}-x_{min}},$$

where $x_{min}$ and $x_{max}$ are computed exclusively from the training cells.

The implemented CNN–BiLSTM model consists of two convolutional layers, a two-layer BiLSTM module, and two fully connected layers. ReLU activation and max-pooling are applied in the convolutional layers, and the final fully connected layer outputs a single regression value. The main architectural and training hyperparameters are summarized in Supplementary Table S6. The convolutional layers operate on each impedance vector to learn local spectral correlations and hierarchical representations that encode degradation-related signatures embedded in the real and imaginary components. By directly leveraging raw spectra, the model preserves electrochemical information across the full frequency domain without relying on equivalent circuit fitting. The extracted features are then processed by BiLSTM layers to capture sequential dependencies in the learned spectral representations. By modeling contextual relationships in both forward and backward directions, the network captures long-range degradation trends inherent to battery aging. This hybrid architecture enables joint learning of frequency-dependent impedance structures and their temporal evolution.

SoH at cycle i is defined as the capacity ratio:

(3) $${SoH}_{i}=\frac{Q_{i}}{Q_{0}},$$

where $Q_{i}$ is the measured capacity at cycle i and $Q_{0}$ denotes the measured capacity of the first cycle for the corresponding cell. EoL is defined as the cycle index $N_{EoL}$ at which SoH declines to 80%. Accordingly, RUL at cycle i is defined as:

(4) $${RUL}_{i}=N_{EoL}-i.$$

Depending on the task configuration, the network output corresponds to either the current SoH for health estimation or the RUL for lifetime prediction. To ensure realistic performance evaluation and mitigate bias arising from cell-to-cell variability, a strict cross-cell validation protocol is adopted in which one cell is sequentially designated as the test set while the remaining cells are used for training, instead of randomly splitting samples into training and test sets. Despite the modest number of cells, the dataset spans different cathode chemistries, cell formats, and cycling conditions, providing a more diverse evaluation setting. This strategy helps mitigate the risk of overfitting.

### 2.3. Salient Feature Analysis

The CNN–BiLSTM model is first trained using the complete impedance spectra consisting of 60 frequency points and then kept fixed during the attribution stage. To evaluate the importance of different spectral components, the impedance spectrum is divided into frequency bands consisting of 10 consecutive frequency points in the ordered 20 kHz–0.02 Hz spectrum. For a given band, the corresponding normalized features in the test cells are neutralized by assigning a value of 0.5. The midpoint represents a neutral, non-informative level after min–max scaling and minimizes artificial bias introduced by extreme values. The perturbed spectra are then fed into the frozen model to obtain modified predictions. The importance of each frequency band is quantified by the increase in prediction error (RMSE) relative to the baseline results obtained from the unperturbed spectra.

## 3. Results and Discussion

### 3.1. Capacity Degradation and Impedance Spectra Evolution

**Figure 1:**
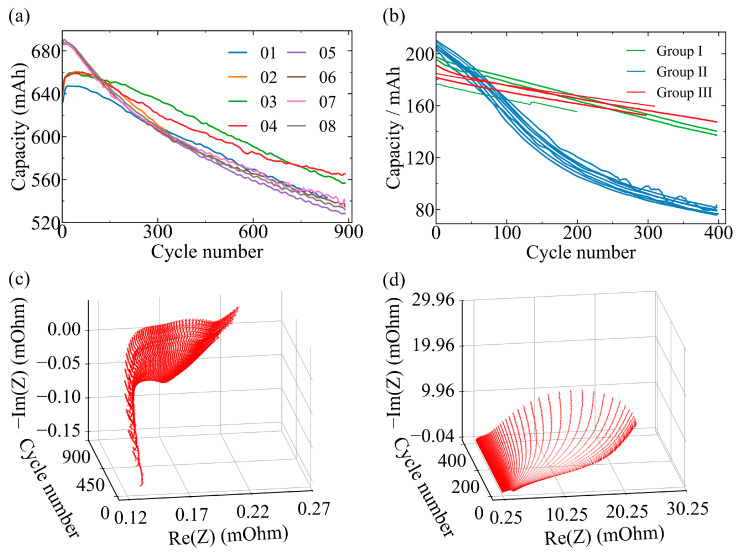
Capacity evolution and impedance spectra of NMC111 and NMC811 cells. (**a**) Capacity evolution of 8 NMC111 cells (NMC111_01-NMC111_08) cycled under identical conditions. (**b**) Capacity evolution of 14 NMC811 cells cycled under three protocols: Group I (green), Group II (blue) and Group III (red). Cells in Group II, cycled to a higher cut-off voltage of 4.3 V, exhibit the fastest capacity degradation. (**c**) Evolution of Nyquist impedance spectra with cycle number for NMC111_01. (**d**) Evolution of Nyquist impedance spectra with cycle number for NMC811_06 at state II.

The capacity degradation of 8 NMC111 cylindrical and 14 NMC811 pouch cells under different cycling conditions is shown in Figure 1a,b. The NMC111 cells exhibit consistent degradation behavior under identical operating conditions. The NMC811 cells display protocol-dependent aging processes, while maintaining good consistency within each group. Cells cycled to a higher upper cutoff voltage to 4.3 V (Group II) degrade significantly faster than those cycled to 4.2 V (Group I and III), suggesting the voltage sensitivity of this system. Cells in Group III, cycled at a higher charge rate (C/6, 2.5–4.2 V), exhibit a degradation trajectory similar to that of the baseline Group I, retaining relatively higher capacities after 200 cycles.

With cycling, the impedance spectra of both NMC111 and NMC811 cells shift towards higher real and imaginary components [49]; however, their spectral evolution differs markedly between the two systems. NMC111 cells exhibit gradual semicircle expansion, whereas NMC811 cells show more complex impedance changes under different operating protocols. Representative EIS spectra for NMC111_01 and NMC811_06 (cycled under high-voltage conditions, state II) are shown in Figure 1c,d. The spectra of NMC811 collected under the other two cycling conditions are presented in Figure S1 in the Supplementary Materials (SM). The accelerated degradation of NMC811 in Group II has been reported to be associated with deep delithiation-induced lattice instability under high-voltage conditions, including structural reconstruction and intensified interfacial reactions [50,51]. Consistently, the impedance spectra exhibit an enhanced low-frequency response, indicating aggravated transport limitations and increased polarization. This mechanistic interpretation is further supported by the salient feature analysis discussed below.

### 3.2. SoH Estimation and RUL Prediction

**Figure 2:**
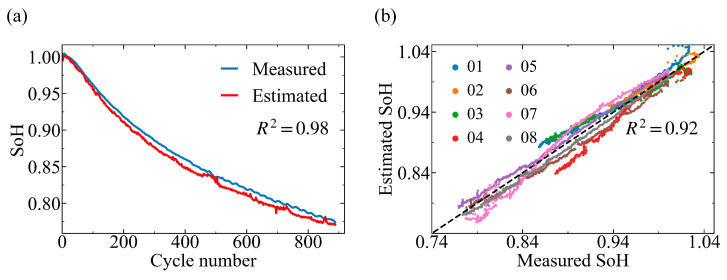
SoH estimation results for NMC111 cells. (**a**) Estimated (red curve) and measured (blue curve) SoH as a function of cycle number for the representative cell NMC111_08. The SoH estimation results for other cells are shown in Figure S2 in the SM. (**b**) The measured SoH against the estimated SoH for all 8 NMC111 cells. The dashed line indicated the ideal prediction. $R^{2}$ is shown in each panel.

We train SoH-estimation models using a CNN-BiLSTM architecture, in which the impedance spectrum from the current cycle is used as model input to estimate the corresponding SoH without explicit knowledge of cycling conditions. Under the strict cross-cell validation protocol, the SoH training set in each fold consists of data from 7 NMC111 cells or 13 NMC811 cells, while the remaining cell is used for testing. Figure 2 shows the SoH estimation results for NMC111 cells. The model accurately estimates the SoH of NMC111, achieving an average $R^{2}$ of 0.92 [Figure 2b]. Given that all NMC111 cells are cycled under identical conditions, these results demonstrate the robustness of the model across cells within a single degradation regime.

**Figure 3:**
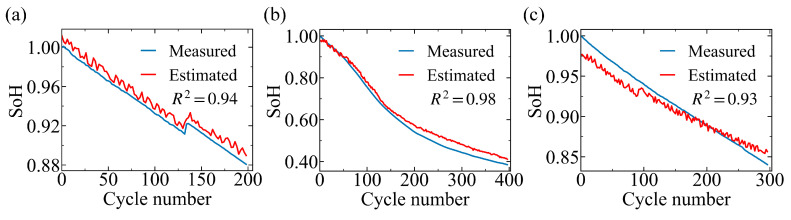
SoH estimation results for representative NMC811 cells cycled under three operating conditions at state II. (**a**–**c**) Results of NMC811_01 (Group I), NMC811_08 (Group II), and NMC811_13 (Group III), respectively. The estimated and measured SoH as a function of cycle number are shown as red and blue curves, respectively. $R^{2}$ is shown in each panel.

We next assess model performance across multiple operating protocols using the NMC811 dataset. Figure 3 shows the SoH estimation results for three representative NMC811 cells selected from each protocol group (i.e., NMC811_01 in Group I, NMC811_08 in Group II, and NMC811_13 in Group III) at state II (15 min rest after fully charging), which gives the highest estimation accuracy among the four states, in agreement with previous findings in the LCO system [43]. A plausible explanation is that EIS measured at high SoC contains more health-sensitive information, whereas the short rest helps mitigate transient polarization and nonequilibrium effects [52], enabling the spectra to more reliably reflect battery health. The results for other cells and states are summarized in Supplementary Table S1. Despite distinct degradation behavior induced by different operating protocols, the model accurately estimates SoH across all groups, achieving an average $R^{2}$ of 0.94 for the NMC811 dataset. Notably, the model successfully reproduces abrupt capacity changes observed in certain cells, such as the capacity recovery at the 132nd cycle in NMC811_01 (Figure 3a). These findings demonstrate that the proposed CNN-BiLSTM architecture based on impedance features achieves accurate and robust performance across chemistries (NMC111 and NMC811), cell formats (cylindrical and pouch formats) and operating protocols for SoH estimation.

**Figure 4:**
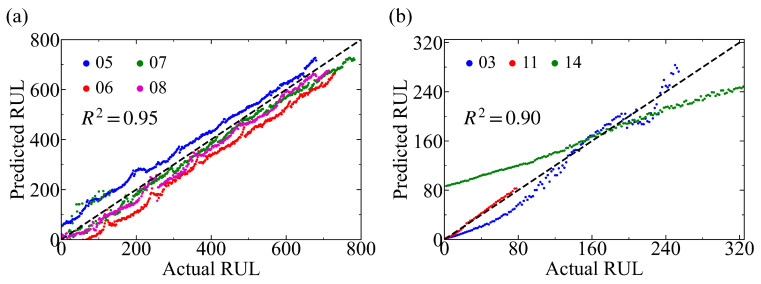
RUL prediction results for NMC111 and NMC811 cells. (**a**) Predicted versus actual RUL for the 4 NMC111 cells that reached their EoL. (**b**) Predicted versus actual RUL for reprehensive NMC811 cells from each group: NMC811_03 (Group I), NMC811_11 (Group II) and NMC811_14 (Group III). The dashed lines in (**a**,**b**) indicate the reference line. The EIS spectra are collected after fully charging followed by a 15 min rest. $R^{2}$ is shown in each panel.

RUL prediction is a critical requirement for advanced battery management systems. Here, we develop models to predict RUL for each battery system using the corresponding EIS spectra. Only cells that reach their EoL are included in the RUL analysis, which yields an RUL dataset of 4 cells for NMC111 and 11 cells for NMC811, including 2 cells in Group I, 8 cells in Group II, and 1 cell in Group III. Accordingly, RUL training is based on 3 NMC111 cells or 10 NMC811 cells. As shown in Figure 4a, the CNN-BiLSTM architecture accurately predicts the RUL of the four NMC111 cells, achieving an average $R^{2}$ of 0.95. The NMC811 system poses greater challenges for RUL prediction due to its shorter cycle life and protocol-dependent degradation behavior. Figure 4b presents representative RUL prediction results for one cell from each group, while the remaining results are provided in Figure S4 in the SM. These results indicate that impedance spectra encode degradation-sensitive features strongly correlated with battery residual lifetime.

### 3.3. Model Benchmarking

To benchmark the proposed framework, we compare the CNN-BiLSTM architecture with several models widely used for battery health prediction, e.g., GPR [42], CNN [53], LSTM [54], and Temporal Convolutional Networks (TCNs) [55]. For a fair comparison, all models are evaluated using the same datasets, preprocessing procedure, and cross-cell validation protocol. In addition, the deep-learning-based models are trained under the same general training settings, including learning rate, batch size, and number of epochs, with only minor architecture-dependent adjustments made when necessary to ensure stable convergence. For both the NMC111 and NMC811 datasets, the CNN–BiLSTM model consistently achieves the best performance in both SoH estimation and RUL prediction, with average $R^{2}$ values exceeding 0.92 and 0.90, respectively. The averaged cross-cell performance of these models for SoH estimation and RUL prediction is listed in Table 2. Performance metrics for individual cells are provided in Tables S2 and S3 in the SM.

For the NMC111 dataset, all models achieve acceptable performance in both SoH estimation and RUL prediction. This can be attributed to the relatively consistent degradation pattern observed under identical cycling conditions, which reduces variability across cells and simplifies the prediction task. In contrast, the NMC811 dataset involves three distinct cycling protocols that induce markedly different degradation processes. As a result, the performance gap between models becomes more pronounced, particularly for the RUL prediction of NMC811 cells. It is worth noting that several NMC811 cells did not reach their EoL (defined as 80% SoH) within the experimental window and were therefore excluded from the training dataset. Despite this limited-data scenario, the CNN–BiLSTM model maintains high predictive accuracy with an average $R^{2}$ of 0.90 for RUL prediction. However, the other models fail to predict the RUL of certain cells, such as the GPR model for NMC811_05 and NMC811_07 and LSTM model for NMC811_02 and NMC811_07. These results demonstrate that our proposed architecture is more robust in capturing protocol-dependent nonlinear aging dynamics.

### 3.4. Interpretation of Impedance Features

**Figure 5:**
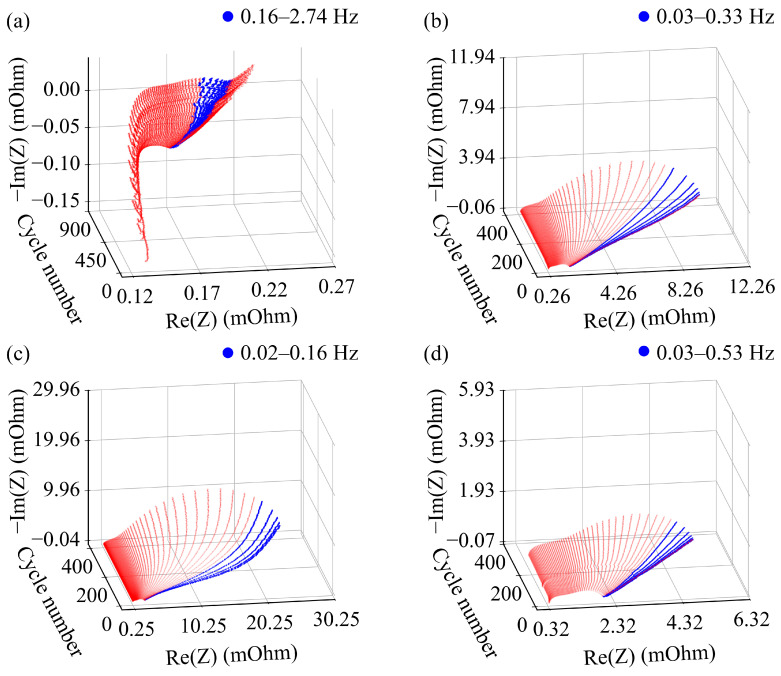
Nyquist impedance spectra with the most influential frequency regions highlighted. The EIS spectra used for feature analysis were collected after fully charging followed by a 15 min rest. The emphasized frequency ranges indicate the most influential bands identified by the CNN–BiLSTM-based band-wise perturbation analysis: (**a**) NMC111 cell, with salient frequencies in the region of 0.16–2.74 Hz. (**b**) NMC811 cell cycled under Group I conditions (2.5–4.2 V, C/40), with salient frequencies in the range of 0.03–0.33 Hz. (**c**) NMC811 cell cycled under Group II conditions (2.5–4.3 V, C/40), with salient frequencies in the range of 0.02–0.16 Hz. (**d**) NMC811 cycled under Group III conditions (2.5–4.2 V, C/6), with salient frequencies in the range of 0.03–0.53 Hz. The spectral components corresponding to the salient frequencies are highlighted in blue in each panel.

To interpret the impedance features learned by the models, we perform a band-wise perturbation analysis based on the CNN-BiLSTM architecture. By quantifying the sensitivity of the prediction to each frequency band, the analysis identifies the most influential regions of the impedance spectrum associated with battery degradation. The spectral components corresponding to the salient frequencies are highlighted on the Nyquist spectra for both battery systems [Figure 5]. For the NMC111 system, the dominant frequencies are located in the low-frequency range of 0.16–2.74 Hz [Figure 5a]. This region is usually associated with diffusion-related processes and concentration polarization in lithium-ion batteries [56,57], suggesting that transport-related limitations play an important role in the aging behavior of the NMC111 system. Combined with the relatively smooth impedance evolution observed for NMC111 during cycling, this result indicates that the degradation signature of NMC111 is dominated more by the gradual accumulation of transport resistance than by abrupt interfacial or structural instability.

For the NMC811 system, the distribution of salient frequencies varies across operating protocols [Figure 5b–d], consistent with their protocol-dependent degradation behavior shown in Figure 1b. Under the baseline cycling condition (Group I, 2.5–4.2 V, C/40), the most influential frequencies are located in the range of approximately 0.03–0.33 Hz [Figure 5b], indicating that slow transport and polarization processes already make a major contribution to the aging response of this Ni-rich system. When the upper cut-off voltage is increased to 4.3 V (Group II), the salient region shifts further toward lower frequencies (0.02–0.16 Hz) [Figure 5c]. Such low-frequency responses are typically associated with long-time-constant processes and transport limitations. In Ni-rich layered cathodes, high-voltage cycling can induce deep delithiation, lattice instability, surface reconstruction, and intensified interfacial reactions [50,51,58], which together increase interfacial heterogeneity and impede ion transport. Meanwhile, these degradation processes can make Li-ion transport increasingly heterogeneous and tortuous, thereby strengthening diffusion-related limitations with longer characteristic times. The further shift of the sensitive region toward lower frequencies therefore suggests that the model mainly captures degradation information related to the gradual accumulation of transport and interfacial limitations under high-voltage operation. As for Group III under higher-rate cycling conditions (2.5–4.2 V, C/6), the dominant frequencies shift slightly toward higher values within the low-frequency domain (approximately 0.03–0.53 Hz) [Figure 5d]. This trend indicates an increased contribution from polarization and charge-transfer-related processes under elevated current density, while transport-related limitations remain important. Compared with Group II, the degradation signature in Group III therefore appears less dominated by extremely slow processes and more influenced by coupled kinetic polarization and transport effects.

## 4. Conclusions

In this work, we develop and experimentally validate a raw-EIS-based AI framework for battery health prognostics using industrial-grade lithium-ion battery cells, including NMC111 cylindrical and NMC811 pouch formats cycled under diverse operating protocols. The proposed CNN–BiLSTM architecture combines convolutional feature extraction with bidirectional temporal modeling to capture nonlinear degradation processes encoded in impedance spectra. The model enables accurate and robust SoH estimation and RUL prediction across both battery systems, outperforming conventional models such as GPR and several neural network models. While simpler models may already provide reliable performance in relatively regular degradation scenarios, the advantage of the proposed CNN–BiLSTM architecture becomes more evident under more heterogeneous conditions requiring stronger robustness.

Beyond predictive performance, salient feature analysis reveals that spectral component with different frequencies exhibit distinct sensitivities under varying chemistries and cycling conditions. For example, the most salient frequencies of NMC111 are in the low-frequency region, consistent with degradation patterns associated with transport- and diffusion-related processes. In contrast, for Ni-rich NMC811, dominant features shift toward even lower frequencies under high-voltage cycling, indicating stronger transport limitations under elevated cut-off voltages. These findings highlight the protocol-dependent nature of impedance-derived degradation signatures.

Overall, our work demonstrates that impedance spectra can serve as broadly applicable degradation descriptors for data-driven battery prognostics. By integrating interpretable spectral analysis with deep-learning-based prediction, the proposed framework offers a practical pathway toward reliable, chemistry-aware battery health monitoring in next-generation BMSs. The robust performance under strict cell-wise evaluation and distinct cycling protocols further supports its applicability under heterogeneous degradation scenarios. Although the present results indicate encouraging generalization potential, performance on completely unseen chemistries remains to be established. Further validation on larger and more diverse datasets will be necessary to assess the robustness of the framework under broader operating conditions and for additional battery chemistries. From a practical perspective, the trained CNN–BiLSTM model imposes a relatively low computational burden at the inference stage, making online prediction feasible in principle. However, deployment in BMSs still requires efficient impedance acquisition, suitable hardware support, and integration with existing workflows. These challenges may be addressed through reduced-frequency acquisition, simplified measurement protocols, and lightweight deployment strategies for embedded systems.

## Figures and Tables

**Table 1:** Overall experimental conditions.

Chemistry	Group	Cell IDs	Cycling Condition	Voltage Window
NMC111	—	01-08	3C charge/1.5C discharge	3.0–4.2 V
NMC811	Group I	01-03	C/40 charge/discharge	2.5–4.2 V
	Group II	04-11	C/40 charge/discharge	2.5–4.3 V
	Group III	12-14	C/6 charge/discharge	2.5–4.2 V

**Table 2:** Comparison of SoH estimation and RUL prediction performance across different models on the NMC111 and NMC811 datasets, including GPR, CNN, LSTM, and TCN. The EIS used for health prognostics are collected after fully charging followed by a 15 min rest. Average $R^{2}$ and RMSE values are reported. All models are trained and evaluated using the same datasets, preprocessing procedure, and cross-cell validation protocol, and the main training settings for the deep-learning-based models are kept consistent to ensure a fair comparison. The best results are highlighted in bold. Detailed results for individual cells are provided in Tables S2 and S3 in the SM.

Model	SoH Estimation				RUL Prediction			
	NMC111		NMC811		NMC111		NMC811	
	$R^{2}$	RMSE	$R^{2}$	RMSE	$R^{2}$	RMSE	$R^{2}$	RMSE
GPR	0.87	0.017	−0.47	0.061	0.91	56.682	0.36	22.346
CNN	0.92	0.014	0.92	0.030	0.91	53.320	0.45	28.030
LSTM	0.92	0.014	0.88	0.032	0.91	58.530	−0.26	23.258
TCN	0.91	0.015	0.87	0.033	0.94	51.396	0.51	20.958
CNN-BiLSTM	0.92	0.013	0.94	0.030	0.95	46.124	0.90	13.960

## Data Availability

All EIS and capacity data supporting this study are available in a public repository (https://github.com/zhihang195/NMC111-811_data, accessed on 14 April 2026).

## Supplementary Materials

The following supporting information can be downloaded at: https://www.mdpi.com/article/10.3390/s26082492/s1, Figure S1: Nyquist impedance spectra of representative NMC811 cells cycled under the other two different operating protocols; Figure S2: SoH estimation results as a function of cycle number for all 8 NMC111 cells; Figure S3: SoH estimation results as a function of cycle number for all NMC811 cells; Figure S4: RUL prediction results for the 11 NMC811 cells; Table S1: Comparison of SoH estimation performance for NMC811 cells at different states using the CNN–BiLSTM model; Table S2: Comparison of SoH estimation performance for all cells across different models; Table S3: Comparison of RUL estimation performance for all cells reaching their EoL across different models; Table S5: Representative impedance data from the 1st EIS measurement of NMC811_01; Table S6: Training hyperparameters of the CNN–BiLSTM model.

## Abbreviations

The following abbreviations are used in this manuscript:

EIS	electrochemical impedance spectroscopy
SoH	state of health
SoC	state of charge
EoL	end of life
RUL	remaining useful life
GPR	gaussian process regression
CNN	convolutional neural network
TCN	temporal convolutional network
LSTM	long short-term memory
BMS	battery management system
