# Supplementary material for: Data-Driven Health Prognostics of NMC Lithium-Ion Batteries via Impedance Spectroscopy Using a Hybrid CNN-BiLSTM Model
Source: Sensors (Basel). 2026 Apr 17;26(8):2492. doi: 10.3390/s26082492 (PMC13119601; doi:10.3390/s26082492)
Supplement: Supplementary file 1 [file sensors-26-02492-s001.zip › sensors-4233832-supplementary.pdf]

Supplementary Materials

Data-Driven Health Prognostics of NMC Lithium-Ion Batteries via Impedance Spectroscopy Using a Hybrid CNN-BiLSTM Model

Zhihang Liu [1,2], Kai Fu [1], Jiahui Liao [1], Ulrich Stimming [3], Donghui Guo [1,2,*] and Yunwei Zhang [1,2,*]

[1] School of Physics, Sun Yat-sen University, Guangzhou 510275, China
[2] Guangdong Provincial Key Laboratory of Magnetoelectric Physics and Devices, Sun Yat-sen University, Guangzhou 510275, China
[3] The Faraday Institution, Quad One, Becquerel Avenue, Harwell Campus, Didcot OX11 0RA, UK

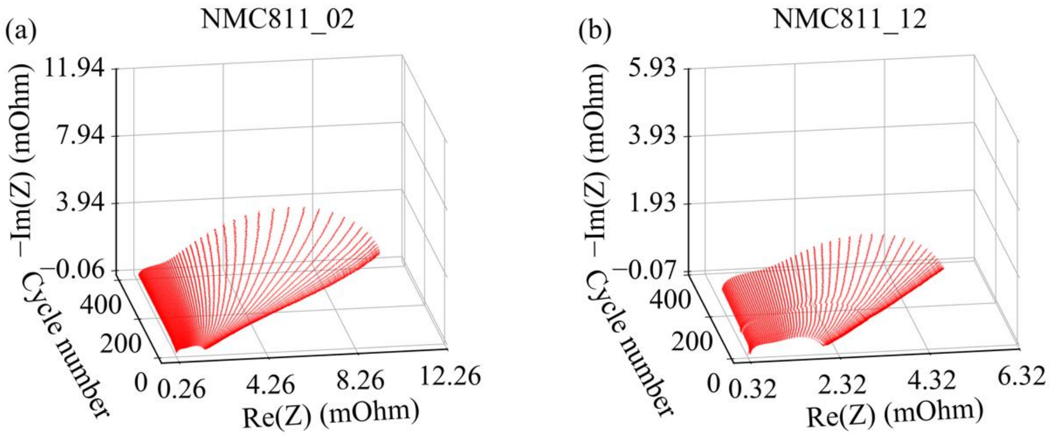

Supplementary Figure S1. Nyquist impedance spectra of representative NMC811 cells cycled under the other two different operating protocols. (a) NMC811_02 in Group I (2.5–4.2 V, C/40). (b) NMC811_12 in Group III (2.5–4.2 V, C/6).

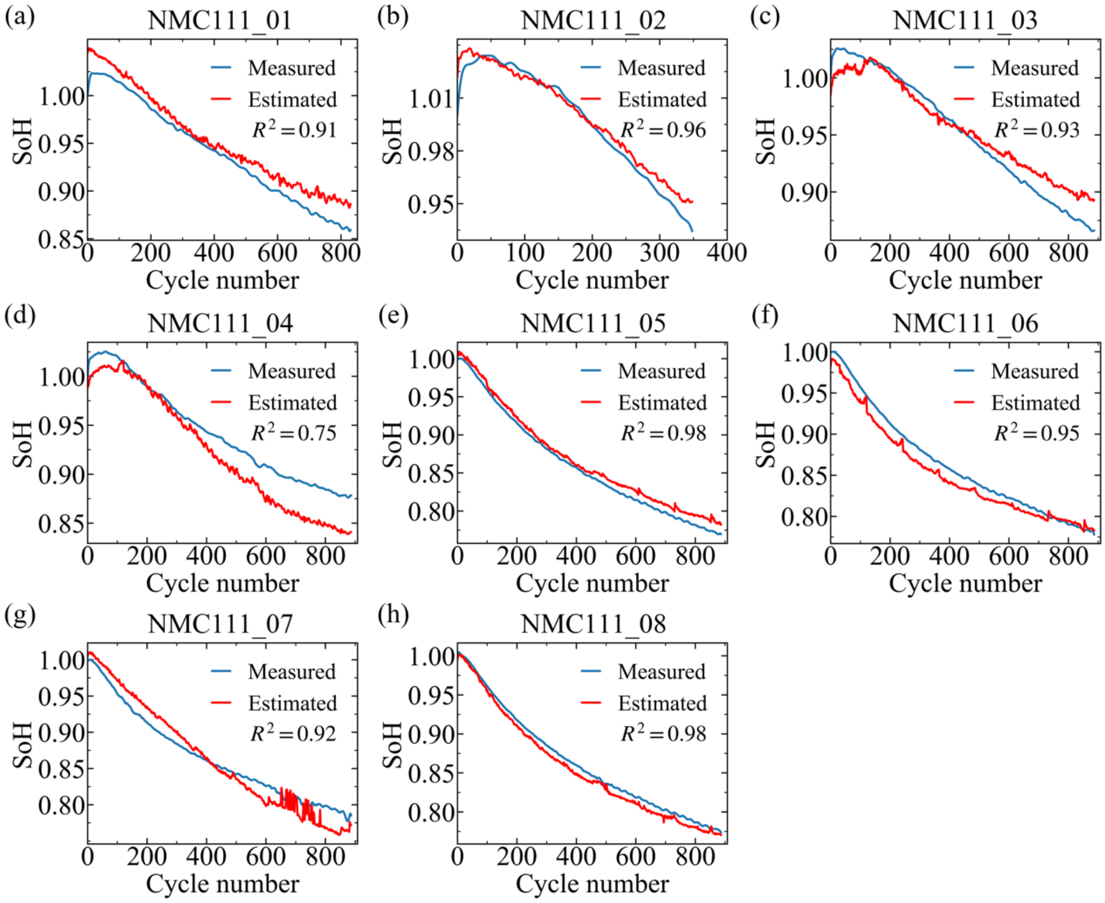

Supplementary Figure S2. SoH estimation results as a function of cycle number for all 8 NMC111 cells. (a–h) correspond to NMC_01-NMC-08. Measured and predicted SoH are shown as blue and red curves, respectively. R^2 value is shown in each panel.

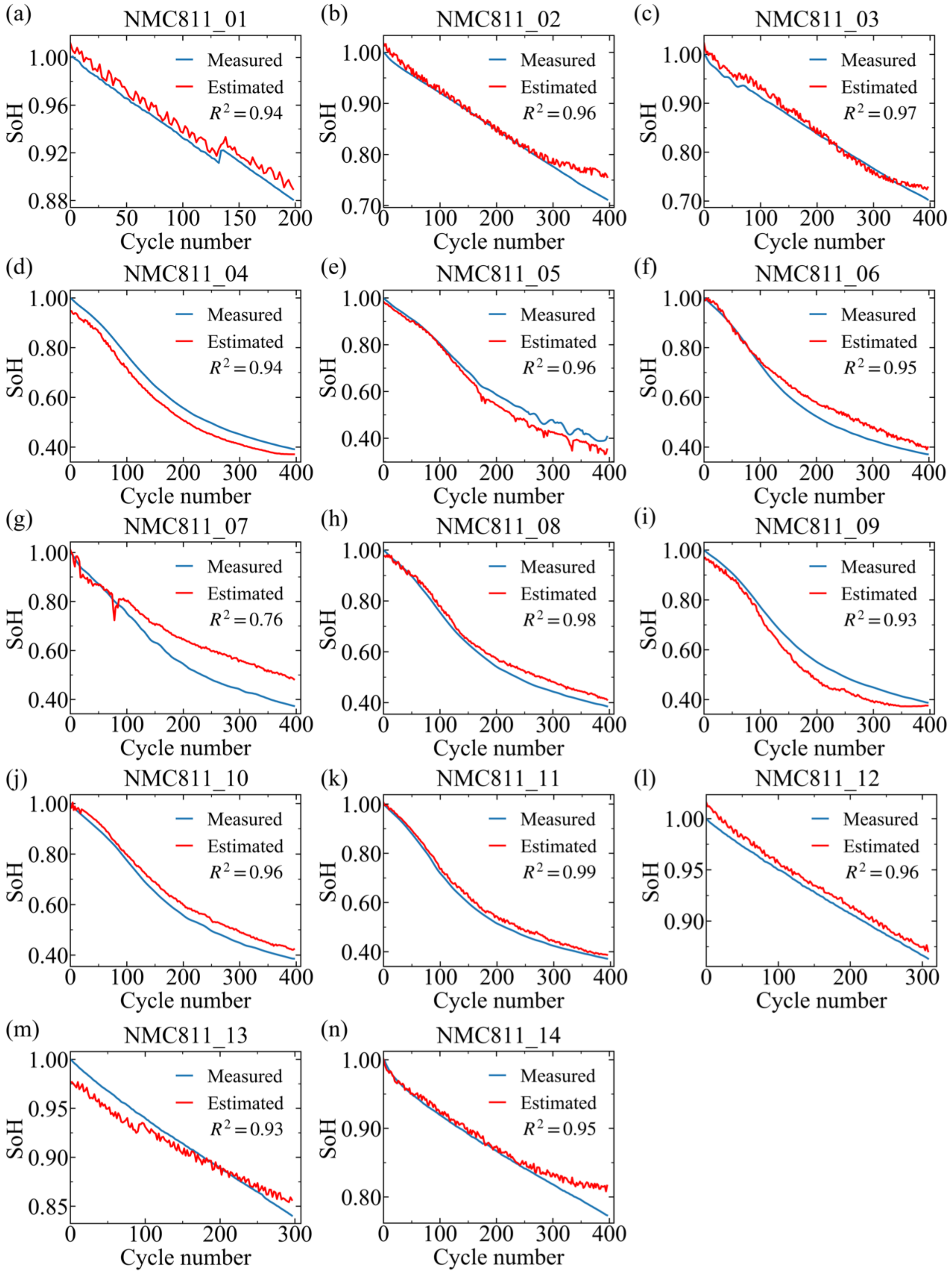

Supplementary Figure S3. SoH estimation results as a function of cycle number for all NMC811 cells. (a–c), (d–k), and (l–n) correspond to cells from Groups I, II, and III, respectively. The measured and estimated SoH are shown as blue and red curves, respectively. R^2 value is shown in each panel.

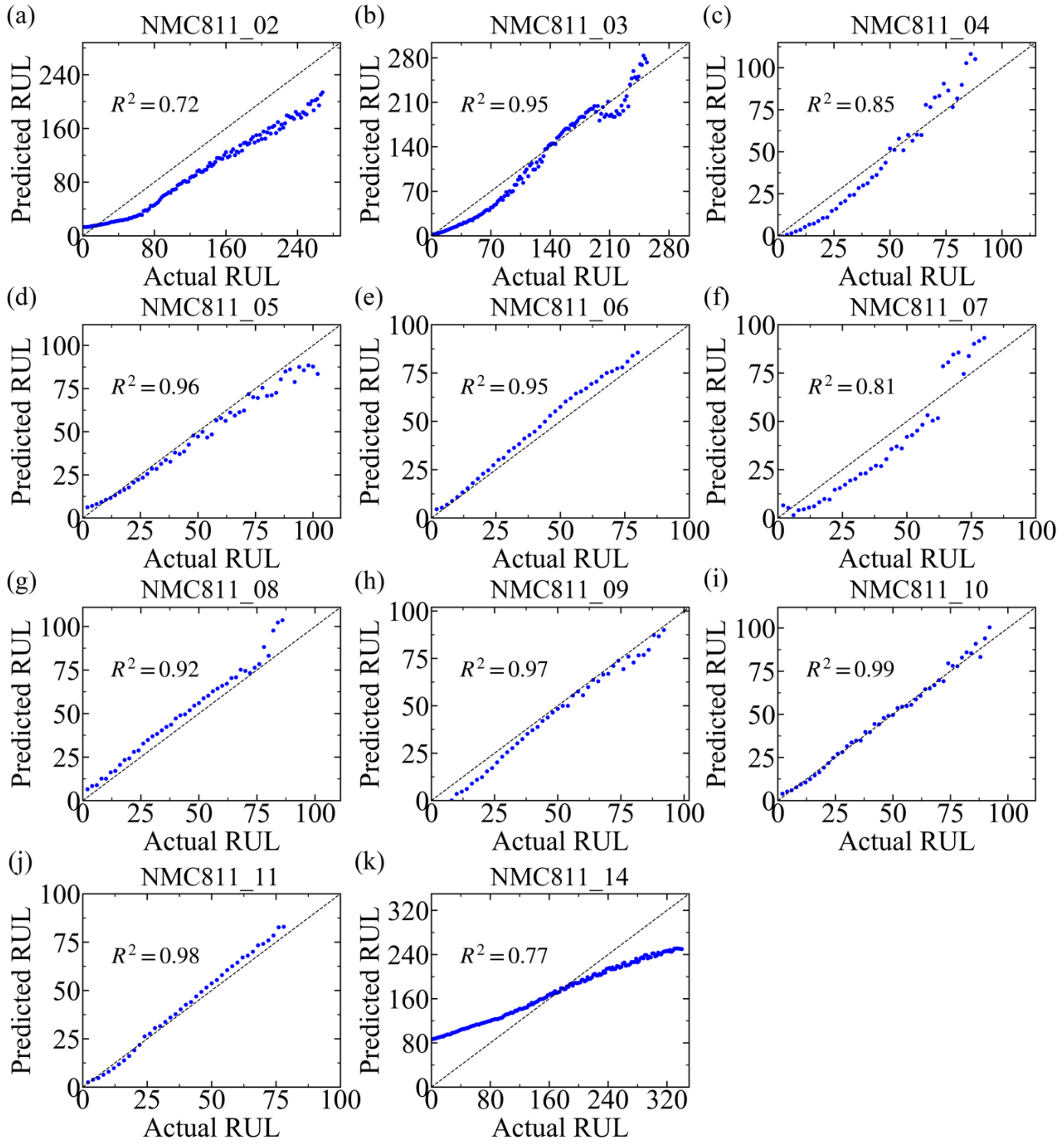

Supplementary Figure S4. RUL prediction results for the 11 NMC811 cells. RUL models are trained using only cells reaching their EoL, defined as 80% SoH. (a–b), (c–j), and (k) correspond to cells from Groups I, II, and III, respectively. R^2 value is shown in each panel. Notably, NMC811_14, the only cell in Group III reaching EoL, exhibits relatively lower prediction accurate ($R^2 = 0.77$) when used as the test cell, due to the limited number of samples representing this degradation regime in the training dataset. In contrast, strong predictive performance is observed for all 8 cells in Group II, where sufficient training samples are available.

Supplementary Table S1. Comparison of SoH estimation performance for NMC811 cells at different states using the CNN–BiLSTM model. Four operating conditions are evaluated: immediately after charging to 100% SoC (state I); after charging to 100% SoC followed by a 15 min rest period (state II); immediately after discharging to 0% SoC (state III); and after discharging to 0% SoC followed by a 15 min rest period (state IV). The highest estimation accuracy is obtained under the State II condition (in bold).

Battery ID	state I		state II		state III		state IV	
	R^2	$RMSE$	R^2	$RMSE$	R^2	$RMSE$	R^2	$RMSE$
01	0.77	0.016	0.94	0.008	0.66	0.020	-2.27	0.061
02	0.11	0.078	0.96	0.017	-1.00	0.117	-0.99	0.116
03	0.97	0.015	0.97	0.013	0.93	0.021	0.59	0.053
04	0.90	0.059	0.94	0.046	0.86	0.070	0.84	0.075
05	0.95	0.042	0.96	0.036	0.95	0.042	0.93	0.051
06	0.98	0.027	0.95	0.043	0.79	0.087	0.10	0.182
07	0.81	0.081	0.76	0.091	0.92	0.053	0.91	0.056
08	0.90	0.060	0.98	0.029	0.93	0.050	0.98	0.028
09	0.95	0.044	0.93	0.050	0.97	0.036	0.94	0.048
10	0.94	0.046	0.96	0.036	0.92	0.054	0.94	0.045
11	0.99	0.023	0.99	0.021	0.99	0.017	0.98	0.027
12	-0.26	0.044	0.96	0.008	-1.94	0.067	-0.58	0.049
13	0.82	0.019	0.93	0.012	0.21	0.040	0.05	0.044
14	0.96	0.011	0.95	0.014	-1.10	0.088	0.49	0.043
**Average**	0.77	0.040	**0.94**	**0.030**	0.36	0.054	0.28	0.063

Supplementary Table S2. Comparison of SoH estimation performance for all cells across different models. All models achieve satisfactory SoH estimation performance on the NMC111 dataset. In contrast, performance decreases on the NMC811 dataset due to the larger variability in degradation mechanisms, with GPR exhibiting the most substantial decline.

**SoH-estimation**	**GPR**		**CNN**		**LSTM**		**TCN**	
	R^2	$RMSE$	R^2	$RMSE$	R^2	$RMSE$	R^2	$RMSE$
NMC111_01	0.70	0.028	0.88	0.018	0.85	0.020	0.87	0.018
NMC111_02	0.92	0.009	0.92	0.008	0.94	0.008	0.95	0.007
NMC111_03	0.82	0.022	0.85	0.020	0.95	0.012	0.83	0.021
NMC111_04	0.78	0.023	0.86	0.018	0.80	0.021	0.76	0.024
NMC111_05	0.95	0.015	0.98	0.010	0.98	0.009	0.97	0.011
NMC111_06	0.91	0.018	0.93	0.016	0.96	0.012	0.92	0.018
NMC111_07	0.92	0.017	0.94	0.015	0.94	0.015	0.95	0.013
NMC111_08	0.99	0.008	0.99	0.005	0.95	0.014	0.98	0.008
**111-Average**	0.87	0.017	0.92	0.014	0.92	0.014	0.91	0.015
NMC811_01	-15.26	0.136	0.95	0.007	0.56	0.021	0.92	0.009
NMC811_02	-0.17	0.090	0.90	0.026	0.47	0.059	0.29	0.070
NMC811_03	0.75	0.042	0.95	0.019	0.97	0.014	0.97	0.015
NMC811_04	0.85	0.072	0.92	0.053	0.93	0.047	0.92	0.055
NMC811_05	0.94	0.045	0.97	0.030	0.95	0.040	0.99	0.023
NMC811_06	0.99	0.023	0.99	0.016	0.89	0.062	0.93	0.051
NMC811_07	-0.15	0.198	0.76	0.090	0.89	0.058	0.79	0.085
NMC811_08	0.96	0.036	0.99	0.020	0.95	0.043	0.97	0.034
NMC811	0.98	0.030	0.95	0.041	0.97	0.031	0.98	0.025

NMC811_09								
NMC811_10	0.81	0.082	0.96	0.040	0.98	0.028	0.99	0.020
NMC811_11	0.99	0.022	0.99	0.021	0.99	0.016	1.00	0.013
NMC811_12	-0.05	0.040	0.70	0.021	0.83	0.015	0.88	0.013
NMC811_13	0.97	0.007	0.91	0.013	0.97	0.008	0.86	0.017
NMC811_14	0.78	0.029	0.88	0.021	0.97	0.010	0.73	0.032
**811-Average**	-0.47	0.061	0.92	0.030	0.88	0.032	0.87	0.033

Supplementary Table S3. Comparison of RUL estimation performance for all cells reaching their EoL across different models. All models achieve accurate RUL prediction on the NMC111 dataset. In contrast, for the NMC811 dataset, the comparison models struggle to capture the diverse degradation patterns, leading to a clear decline in generalization performance.

**RUL-prediction**	**GPR**		**CNN**		**LSTM**		**TCN**	
	R^2	$RMSE$	R^2	$RMSE$	R^2	$RMSE$	R^2	$RMSE$
NMC111_05	0.94	48.904	0.93	50.612	0.94	49.306	0.94	49.114
NMC111_06	0.77	100.390	0.74	107.950	0.82	89.596	0.92	60.656
NMC111_07	0.95	52.650	0.97	39.108	0.93	60.706	0.91	68.210
NMC111_08	0.99	24.786	0.99	15.608	0.97	34.514	0.98	27.602
**111-Average**	0.91	56.682	0.91	53.320	0.91	58.530	0.94	51.396
NMC811_02	0.96	14.910	0.38	60.730	0.03	73.900	0.22	68.158
NMC811_03	0.74	37.132	0.82	30.822	0.81	31.300	0.93	19.450
NMC811_04	0.79	11.778	0.20	23.184	0.76	11.528	0.50	18.398
NMC811_05	-1.59	47.390	0.69	16.416	0.97	4.528	0.88	10.132
NMC811_06	0.89	7.620	0.24	20.132	0.97	3.768	0.96	4.694
NMC811_07	-2.16	41.046	-0.99	32.578	-11.09	72.246	-2.48	43.092
NMC811_08	0.93	6.562	0.73	12.984	0.95	4.954	0.91	7.284
NMC811_09	0.96	5.182	0.96	5.148	0.98	3.618	0.94	6.464
NMC811_10	0.91	8.080	0.89	8.998	0.98	3.742	0.98	3.800
NMC811_11	0.93	5.990	0.87	8.246	1.00	1.262	0.98	3.454
NMC811_14	0.62	60.116	0.18	89.086	0.78	44.998	0.78	45.622
**811-Average**	0.36	22.346	0.45	28.030	-0.26	23.258	0.51	20.958

Supplementary Table S4. Initial capacities (Q_0) for NMC cells used in this study.

Battery ID	Q_0/mAh	Battery ID	Q_0/mAh
NMC111_01	632	NMC811_04	209
NMC111_02	639	NMC811_05	205
NMC111_03	643	NMC811_06	207
NMC111_04	644	NMC811_07	203
NMC111_05	686	NMC811_08	206
NMC111_06	687	NMC811_09	210
NMC111_07	687	NMC811_10	205
NMC111_08	687	NMC811_11	205
NMC811_01	176	NMC811_12	185
NMC811_02	197	NMC811_13	182
NMC811_03	195	NMC811_14	191

Supplementary Table S5. Representative impedance data from the 1st EIS measurement of NMC811_01.

Cycle number	Frequency	Z	Z_{re}	Z_{im}	Phase
1	20004.45	1.724005	1.722853	0.062991	2.093917
1	15829.13	1.731289	1.731158	0.021292	0.704661
1	12516.7	1.744606	1.744531	-0.01617	-0.53116
1	9909.442	1.758359	1.757618	-0.05104	-1.6633
1	7835.48	1.776359	1.774495	-0.08136	-2.62504
1	6217.246	1.79928	1.795903	-0.11019	-3.51101
1	4905.291	1.825311	1.820091	-0.13794	-4.33397
1	3881.274	1.856756	1.849617	-0.16266	-5.02594
1	3070.983	1.88808	1.879082	-0.1841	-5.59568
1	2430.778	1.925879	1.915095	-0.20352	-6.06624
1	1923.154	1.963167	1.950856	-0.21952	-6.42006
1	1522.436	2.002983	1.989205	-0.23453	-6.72408
1	1203.845	2.04444	2.029389	-0.24762	-6.95659
1	952.8659	2.08369	2.067758	-0.25718	-7.08974
1	754.2756	2.127125	2.110029	-0.26915	-7.26914
1	596.7186	2.170298	2.152506	-0.27732	-7.34137
1	471.9634	2.212737	2.19393	-0.28788	-7.47546
1	373.2086	2.257409	2.238002	-0.29537	-7.51837
1	295.4728	2.301006	2.280755	-0.3046	-7.60708
1	233.8774	2.346858	2.325653	-0.31477	-7.70788
1	185.0592	2.391393	2.368859	-0.32751	-7.87167
1	146.3582	2.438023	2.414258	-0.33958	-8.0065
1	115.778	2.48683	2.461422	-0.35458	-8.19735

1	91.6721	2.536701	2.50912	-0.37306	-8.4568
1	72.51701	2.588632	2.558533	-0.3936	-8.7458
1	57.36816	2.644233	2.611405	-0.41537	-9.03774
1	45.3629	2.703668	2.666847	-0.44469	-9.46675
1	35.93134	2.767654	2.7263	-0.47665	-9.91702
1	28.40909	2.837913	2.791441	-0.51147	-10.3831
1	22.48202	2.915997	2.862963	-0.55361	-10.9441
1	17.79613	2.998975	2.938543	-0.59901	-11.5217
1	14.06813	3.094892	3.025618	-0.65115	-12.1455
1	11.1448	3.199675	3.121822	-0.70153	-12.6651
1	8.81772	3.318601	3.231198	-0.75662	-13.179
1	6.975446	3.447639	3.350473	-0.81274	-13.6351
1	5.517303	3.589755	3.482397	-0.87135	-14.0479
1	4.369407	3.761833	3.646992	-0.92241	-14.1938
1	3.456858	3.918439	3.799613	-0.95765	-14.1462
1	2.735469	4.114721	3.993899	-0.9898	-13.9191
1	2.160537	4.289379	4.169992	-1.00496	-13.5498
1	1.709518	4.468717	4.360205	-0.97879	-12.6522
1	1.353517	4.645306	4.548351	-0.94412	-11.7266
1	1.070792	4.796077	4.714132	-0.88279	-10.6066
1	0.847343	4.930471	4.865226	-0.79945	-9.33146
1	0.670716	5.04652	4.995632	-0.71486	-8.14356
1	0.530668	5.138486	5.099254	-0.63376	-7.08471
1	0.419756	5.201552	5.171474	-0.55857	-6.16463
1	0.331825	5.246193	5.223624	-0.48609	-5.31644
1	0.262605	5.276148	5.25942	-0.41981	-4.56371
1	0.207912	5.299421	5.287185	-0.35992	-3.89433
1	0.164515	5.319654	5.310737	-0.30788	-3.31788
1	0.130065	5.342072	5.335399	-0.26692	-2.86399
1	0.103094	5.366186	5.360782	-0.24076	-2.57155
1	0.08153	5.389824	5.384841	-0.23171	-2.46392
1	0.064426	5.405757	5.400551	-0.2372	-2.51488
1	0.05102	5.40962	5.403465	-0.25799	-2.73349
1	0.040421	5.395677	5.388512	-0.27796	-2.9529
1	0.031919	5.374509	5.366815	-0.28748	-3.0662
1	0.025282	5.357846	5.35021	-0.28595	-3.05938

Supplementary Table S6. Training hyperparameters of the CNN–BiLSTM model.

Parameters	Setting
convolutional layers	2
kernel size	1 × 2

BiLSTM layers	2
hidden size	64
activation function	ReLU
batch size	64
learning rate	0.001
epochs	150
